# Spatial distribution of traffic induced noise exposures in a US city: an analytic tool for assessing the health impacts of urban planning decisions

**DOI:** 10.1186/1476-072X-6-24

**Published:** 2007-06-21

**Authors:** Edmund Yet Wah Seto, Ashley Holt, Tom Rivard, Rajiv Bhatia

**Affiliations:** 1 Environmental Health Sciences, School of Public Health, University of California, Berkeley, CA, USA; 2 Environmental Science Policy Management, College of Natural Resources, University of California, Berkeley, CA, USA; 3 Department of Public Health, San Francisco, CA, USA; 4 Department of Medicine, University of California, San Francisco, USA

## Abstract

**Background:**

Vehicle traffic is the major source of noise in urban environments, which in turn has multiple impacts on health. In this paper we investigate the spatial distribution of community noise exposures and annoyance. Traffic data from the City of San Francisco were used to model noise exposure by neighborhood and road type. Remote sensing data were used in the model to estimate neighborhood-specific percentages of cars, trucks, and buses on arterial versus non-arterial streets. The model was validated on 235 streets. Finally, an exposure-response relationship was used to predict the prevalence of high annoyance for different neighborhoods.

**Results:**

Urban noise was found to increase 6.7 dB (p < 0.001) with 10-fold increased street traffic, with important contributors to noise being bus and heavy truck traffic. Living along arterial streets also increased risk of annoyance by 40%. The relative risk of annoyance in one of the City's fastest growing neighborhoods, the South of Market Area, was found to be 2.1 times that of lowest noise neighborhood. However, higher densities of exposed individuals were found in Chinatown and Downtown/Civic Center. Overall, we estimated that 17% of the city's population was at risk of high annoyance from traffic noise.

**Conclusion:**

The risk of annoyance from urban noise is large, and varies considerably between neighborhoods. Such risk should be considered in urban areas undergoing rapid growth. We present a relatively simple GIS-based noise model that may be used for routinely evaluating the health impacts of environmental noise.

## Background

Landuse and transportation development policies have significant effects on health and the environment [1]. While development is often associated with increased use of automobiles, which can adversely affect physical activity [2], injuries [3,4], and air pollution-related health [5-9], good landuse and transportation policies can potentially reduce these adverse effects, and promote wellness through increased access, mobility, and walking.

Automobile traffic is one of the primary sources of community noise. Recent reviews by Stansfeld and Matheson [10], Shield and Dockrell [11], and Passchier-Vermeer [12] document the relationships between noise exposure and annoyance [13-19], sleep disturbance [20-23], hypertension and cardiovascular disease [24-28], mental disorder [29-31], and children's cognition, including speech intelligibility, reading comprehension, memory, motivation, attention, problem-solving, and performance on standardized tests [15]. These effects may relate not only to the intensity of noise, but also its temporal variation, frequency range, perceived threat or lack of control associated with the noise, whether or not adaptation to the noise occurs, and the degree of interaction with other stressors [10,16,32,33]. Moreover, studies suggest that noise is just one of many physical and psychosocial stressors that work together to affect the socioemotional development of children living in poverty [34], as well as the functional health and well-being of older adults [35]. Of all health effects associated with noise, the dose-response relationship between community noise and annoyance is the most developed. High annoyance to noise is typically determined via questionnaires. Despite it being a relatively subjective measure, its association with community noise has been found to be fairly consistent across multi-national studies [12].

Generally it has been recognized that environmental hazards in urban areas disproportionately affect low-income people [36]. However, few studies have documented the inequalities in noise exposures that exist as a result of land use and transportation development policies. European studies have found that higher noise exposures are associated with low income [37], and that traffic noise adversely impacts rates of physical activity [38]. Yet those that are more affluent may be more likely to complain about environmental noise [39]. While the aforementioned studies provide some evidence for the inequities in noise exposures in Europe, we are aware of no assessments of urban noise done in recent years for any US city. This is partly due to different attitudes towards environmental noise and the lack of federal-funded noise research and regulation [40]. Hence, exposures to environmental noise are poorly understood in the US. As a first step to understanding noise inequities it is necessary to understand the spatial variation in noise exposures that exist in US urban areas.

In recent years, the City of San Francisco has received increasing numbers of noise complaints due to the juxtaposition of new residential development with existing commercial and industrial land use. This has motivated the need for noise and annoyance maps to better inform future redevelopment. Such a map would also inform potential inequalities in noise exposures that may occur between the city's diverse communities that include high-rise financial districts, multi-million dollar residential, multiple ethnic, public housing areas, and redeveloping industrial neighborhoods. Due to the demand for further housing, city planners may use such maps to better balance pressure to build more high-density housing, while at the same time trying to consider issues of community preservation and the larger socio-environmental implications of their decisions.

This paper describes a quantitative assessment of the spatial distribution of transportation-related noise exposures, and their impact on population annoyance for neighborhoods in San Francisco. We present a geographic information system (GIS) -based noise and annoyance model that relies on the city's extensive traffic count database, as well as an analysis of aerial photography to determine the proportion of different types of vehicles in different neighborhoods. We explore and discuss the implications of spatial variation in neighborhoods at risk for environmental noise exposure.

## Results

**Figure 1:**
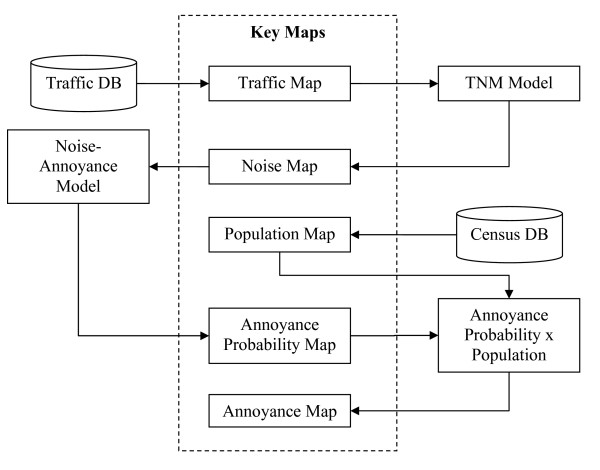
Flow diagram of GIS Traffic Noise Annoyance Model.

A diagram of the steps in the GIS model from traffic data to noise to estimates of high annoyance is presented in figure 1. Along the pathway, maps were created to better understand the role that environmental conditions play in community health for each neighborhood.

### Characteristics of the measured traffic

**Figure 2:**
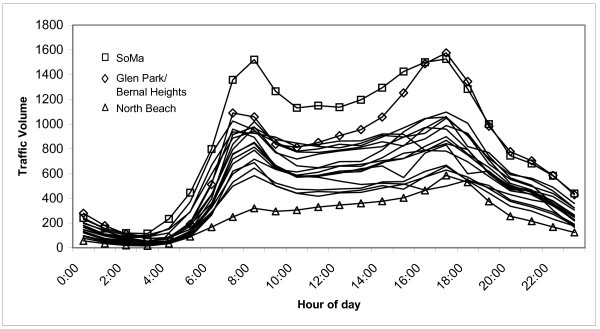
Average hourly traffic for different San Francisco neighborhoods (three neighborhoods with different temporal characteristics from the others are labeled).

The first step in the model consisted of an assessment of community traffic. Most neighborhoods followed a consistent daily temporal pattern with rush hour peaks between 06:00 – 10:00 and 15:00 – 19:00 hr (figure 2). On average, the rush hour periods accounted for 50% of the daily traffic volume. Based on the city-wide average time series, 73% of 24-hour traffic occurred during the daytime hours (07:00 – 19:00), 16% occurred during the evening hours (19:00 – 23:00), and 11% occurred during the nighttime hours (23:00 – 07:00). Traffic data from counted streets are summarized by neighborhood and by arterial versus non-arterial streets in Table 1. The table also presents the differences in neighborhood with respect to the percentage of vehicle traffic that were medium trucks, heavy trucks, or buses.

**Table 1:** Measured traffic counts from pneumatic tube counters and vehicle type percentages determined from remote sensing for arterial and non-arterial streets by neighborhood.

		24-hr Traffic (1,000s)				Vehicle type (%)					
		Non-arterial		Arterial		Non-arterial			Arterial		

Neighborhood	No. street segments	Mean	25–75%ile	Mean	25–75%ile	Medium Truck	Heavy Truck	Bus	Medium Truck	Heavy Truck	Bus

Bayview/Hunter's Point	86	6	2–7	16	12–22	6	4	2	6	3	3
Central West	161	5	3–7	19	11–26	2	1	2	2	1	0
Chinatown	15	11	11–11	17	10–23	2	1	4	1	2	4
Downtown/Civic Center	53	14	9–17	25	20–28	4	4	1	2	2	4
Excelsior/Visitation Valley/Crocker/Outer Mission	188	4	1–5	12	7–15	0	6	1	0	1	4
Financial District	114	15	8–19	31	12–45	3	1	5	3	2	8
Glen Park/Bernal Heights	80	2	1–3	20	9–25	7	2	1	3	1	5
Haight Ashbury	60	6	3–7	29	13–37	5	6	0	3	0	0
Inner Mission	119	9	1–13	23	14–31	4	4	2	10	1	7
Lakeshore	96	7	1–11	21	12–31	2	2	4	2	0	0
Nob Hill/Russian Hill/Pacific Heights/Marina	133	7	2–8	22	14–30	9	5	0	2	1	5
North Beach	43	6	3–8	19	15–18	6	4	3	1	5	4
Northwest	181	4	2–6	25	13–28	5	0	3	3	0	2
Potrero Hill	73	5	2–7	19	12–23	9	2	6	5	5	3
South of Market	106	17	10–24	24	17–30	3	6	2	1	2	9
Twin Peaks/Diamond Heights/Oceanview	199	5	1–4	13	9–15	5	0	2	4	1	2
Upper Market/Noe Valley	126	4	1–5	14	11–19	8	3	0	6	2	5
Western Addition	166	10	4–10	30	20–44	7	4	2	2	3	0
											
All	1,999	7	2–9	20	11–25	5	3	2	3	2	4

### Analysis of extrapolated traffic by neighborhood

Using the neighborhood-specific arterial and non-arterial traffic averages in Table 1 we extrapolated traffic counts to the remaining uncounted streets. The number of arterial and non-arterial street segments, and cumulative traffic across all streets by neighborhood are presented in Table 2. The highest noise levels were found in the South of Market (SoMa) neighborhood.

**Table 2:** Extrapolated traffic and noise outcomes in dB for the entire city by neighborhood.

	No. street segments		Total 24-hr traffic (1,000s)		Noise level (dB Ldn)		
Neighborhood	Non-arterial	Arterial	Non-arterial	Arterial	Non-arterial	Arterial	Mean (SD)

Bayview/Hunter's Point	1,226	170	7,571	2,683	66	70	66 (1.6)
Central West	1,400	286	7,209	5,512	62	67	63 (2.3)
Chinatown	155	34	1,637	578	66	69	67 (1.6)
Downtown/Civic Center	291	63	4,161	1,550	69	71	69 (1.0)
Excelsior/Visitation Valley/Crocker/Outer Mission	1,567	366	6,925	4,328	64	66	64 (1.5)
Financial District	343	93	5,077	2,841	67	72	68 (2.6)
Glen Park/Bernal Heights	747	77	1,662	1,553	60	69	61 (2.8)
Haight Ashbury	222	61	1,317	1,785	65	69	66 (2.2)
Inner Mission	577	130	5,344	2,995	67	71	68 (2.8)
Lakeshore	407	134	2,903	2,787	63	66	64 (2.5)
Nob Hill/Russian Hill/Pacific Heights/Marina	959	135	6,672	2,958	66	69	67 (1.5)
North Beach	486	43	2,849	805	65	70	65 (1.6)
Northwest	1,379	294	6,012	7,267	61	68	63 (3.0)
Potrero Hill	439	44	2,339	815	65	71	65 (2.1)
South of Market	569	126	9,829	2,994	70	70	70 (1.5)
Twin Peaks/Diamond Heights/Oceanview	1,379	243	6,370	3,060	61	66	62 (2.6)
Upper Market/Noe Valley	709	213	2,703	3,043	63	68	64 (2.6)
Western Addition	601	122	5,750	3,670	67	71	68 (2.6)
							
All	13,456	2,634	86,330	51,224	64	68	65 (3.3)

### Estimated noise levels

**Figure 3:**
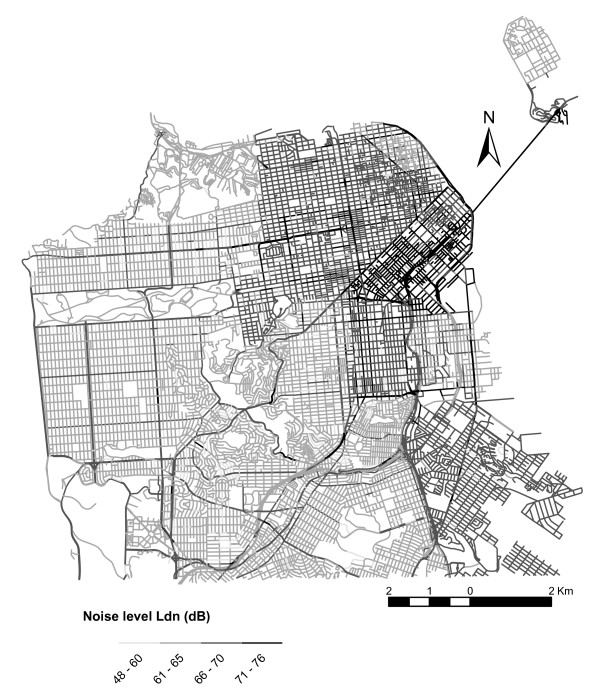
Predicted noise levels along city streets.

**Figure 4:**
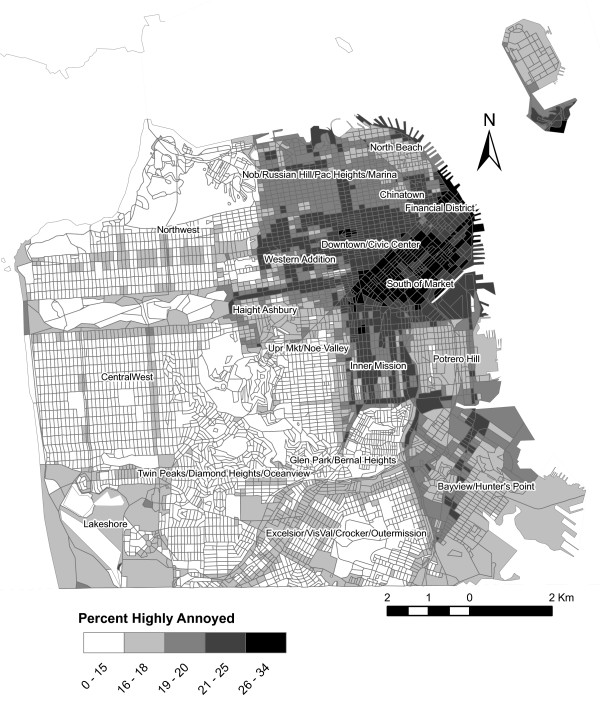
Spatial distribution of the risk of high annoyance to traffic noise.

Traffic-induced noise levels estimated from the Federal Highway Administration's (FHWA) Traffic Noise Model (TNM) 2.5 model [41] are shown in figure 3, and summarized by neighborhood in Table 3. Because the probability of high annoyance is exponentially related to noise (Figure 4), it mimics the general spatial pattern of community noise levels (Figure 3). Hence, the noise levels were greatest in SoMa and so too were the risks of annoyance.

**Table 3:** Population characteristics for each neighborhood, predicted noise, numbers of highly annoyed.

Neighborhood	Population (1,000s)	Population per 100 m^2^	Noise level (dB Ldn)	Highly annoyed (1,000s)	Prevalence of highly annoyed	Highly annoyed per 100 m^2^
Bayview/Hunter's Point	33	0.27	66	6	18%	0.05
Central West	100	0.56	63	13	13%	0.07
Chinatown	10	2.88	67	2	20%	0.54
Downtown/Civic Center	40	2.37	69	9	23%	0.55
Excelsior/Visitation Valley/Crocker/Outer Mission	98	0.78	64	15	15%	0.12
Financial District	4	0.21	68	1	25%	0.04
Glen Park/Bernal Heights	31	0.84	61	4	13%	0.10
Haight Ashbury	23	1.08	66	4	17%	0.19
Inner Mission	48	1.24	68	10	21%	0.25
Lakeshore	18	0.19	64	3	17%	0.03
Nob Hill/Russian Hill/Pacific Heights/Marina	82	1.23	67	16	20%	0.24
North Beach	12	0.68	65	2	17%	0.11
Northwest	85	0.53	63	12	14%	0.07
Potrero Hill	11	0.27	65	2	18%	0.04
South of Market	22	0.38	70	6	27%	0.10
Twin Peaks/Diamond Heights/Oceanview	58	0.51	62	7	12%	0.06
Upper Market/Noe Valley	51	0.98	64	8	16%	0.15
Western Addition	51	1.30	68	10	20%	0.26
						
All	775	0.64	65	128	17%	0.11

### Distribution of the general population and those highly annoyed by noise

**Figure 5:**
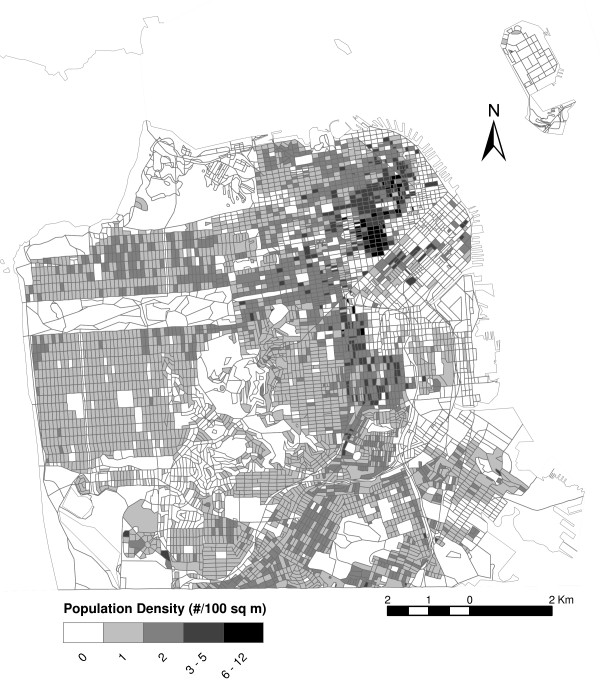
Population density in 2000 by census block.

**Figure 6:**
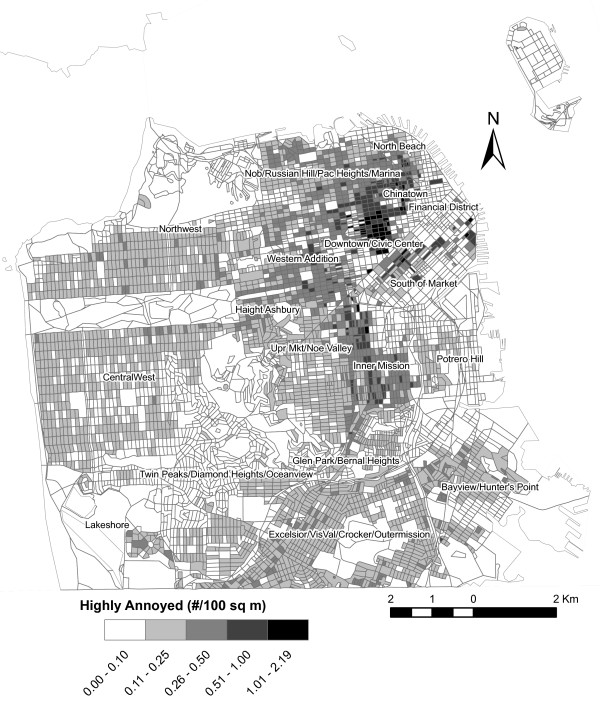
Spatial distribution of the density of high annoyance.

Figure 5 shows the distribution of the population by census block in the city. The greatest population densities are located in Chinatown and Downtown/Civic Center. Multiplying the population in each census block by the probability of being highly annoyed by traffic noise (Figure 4) results in the estimate of highly annoyed (Figure 6 and Table 3). The spatial distribution of risk (probability) of high annoyance differs dramatically from the resulting population density of highly annoyed. Although the risk was greatest in SoMa, few people lived there in the year 2000, and hence, only small pockets of highly annoyed existed within that neighborhood. In contrast the highest densities of highly annoyed were in Downtown/Civic Center. Of the approximately 775,000 people living in the city, we estimated that 17% had the potential to be highly annoyed by noise.

### Field validation of the traffic noise model

**Figure 7:**
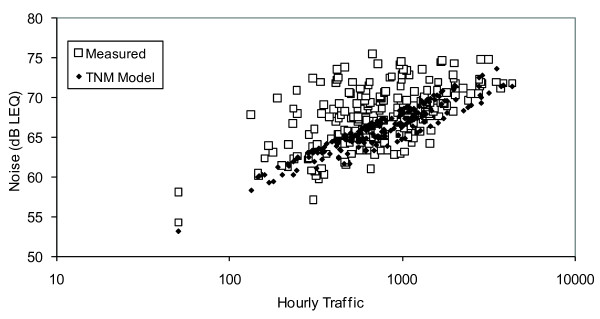
Relationship between time-of-day adjusted hourly traffic counts (log scale) and field measured noise (squares), and predicted noise from the TNM model (diamonds).

Noise measurements were obtained at 235 field sites where traffic count data existed. The relationship between traffic counts (log scale) and measured noise L_Aeq _is shown in figure 7. Linear regression models were used to assess the relationship between measured noise to log transformed traffic counts, and the effect neighborhood-level adjustments for time of day and truck and bus percentages. A simple model of noise using only log hourly traffic based on a weighting factor (w(k, t)), which accounts for neighborhood-specific hourly differences resulted in estimated noise increases of 6.7 dB for a 10-fold increase in traffic (95 % CI: 5.4–7.9, and R^2 ^= 0.33). A second model that adjusted for neighborhood differences in vehicle makeup resulted in an improved fit (R^2 ^= 0.37) with important contributors being increases of 0.3 dB (p < 0.001) and 0.2 dB (p < 0.07) when the neighborhood traffic makeup is increased by 1-percent for buses or heavy trucks, respectively. This supports the need for accounting for noise contributions from different sources of traffic.

Figure 7 also shows the relationship between traffic counts and noise predicted by the TNM model. The relationship between TNM modeled noise versus measured noise was fit to a linear regression with an intercept of 21.7 (95 % CI: 13.4–30.1) and slope of 0.70 (95 % CI: 0.57–0.82) (R^2 ^= 0.34), with errors that were normally distributed. This suggests that the TNM model underestimates noise in environments < 73 dB, and overestimates otherwise. An analysis of potential spatial-temporal factors related to the errors suggests that the error is only weakly related to the time of day field sampling occurred. In addition, we mapped the error by geographic location, and found a mix of both positive and negative errors in each neighborhood, which indicate that errors are not spatially autocorrelated. Hence, the TNM model does not tend to under or over predict noise in any particular neighborhood. These findings suggest that both the time of day weighting factors and neighborhood-specific inputs in the TNM model are important and appropriate.

## Discussion

The objective of this paper was to describe spatial variation in environmental noise exposures and estimate high annoyance to traffic noise for neighborhoods in San Francisco. In the analyses it was important to account for differences between neighborhoods and street types. First, we found that traffic varied considerably across the city both between and within neighborhoods (Table 1). Since the neighborhoods varied in size, so too did the number of street segments within them. However, the number of streets within a neighborhood was not correlated with traffic (arterial or non-arterial). This reflected the commercial land-use and public services of the city which creates transportation demand, for instance high non-arterial traffic in Chinatown, Civic Center, the Financial District, and SoMa. The traffic data also clearly demonstrated the importance of arterials as major transit pathways within and between neighborhoods in the city. On average, counts on arterials were 2.7 times those of non-arterials, and were consistently higher than non-arterials in all neighborhoods.

The temporal pattern of traffic volumes were also neighborhood-dependent (Figure 2). Based on the measured counts, traffic was on average 72% greater in SoMa than the city average, despite the population of SoMa ranking only thirteenth highest amongst the 18 neighborhoods in the city. SoMa serves as a major thoroughfare that serves the downtown Financial District and Civic Center (themselves high traffic areas), the baseball park, and the Moscone Convention Center with on and off-ramps for the three major freeways. SoMa experienced the greatest fluctuations during rush hours because most commuters enter and leave the city via this neighborhood. The other noticeable peak was in Glen Park/Bernal Heights, which like SoMa was influenced by nearby freeways. In contrast, North Beach is a relatively isolated community in the northeast corner of the city, far from the freeways, and thus had the lowest rush hour peaks.

We explored the relationship between traffic and noise using two methods. The first relied on our own field collected noise measurements. Again, neighborhood-level differences were important. The fit of regression models relating noise measurements to traffic measurements using city-wide averages to adjust for time of day improved more than two-fold with the addition of neighborhood-level adjustments for time of day. The relationship was further improved by neighborhood-specific adjustments for vehicle makeup. We found differences in buses percentages to be particularly important, which varied from 0 to 6.5% between neighborhoods. The importance of vehicle makeup is related to empirical data in TNM, which indicate that a bus is equivalent in noise to 12 automobiles, a medium truck is equivalent to nine automobiles, and a heavy truck is equivalent to over 22 automobiles. While buses may be noisier than cars, they may offer substantial benefits to neighborhoods that have bus service. Moreover, the city is currently in the process of replacing its bus fleet with cleaner and quieter buses, which may negate some of the adverse noise effects.

**Figure 8:**
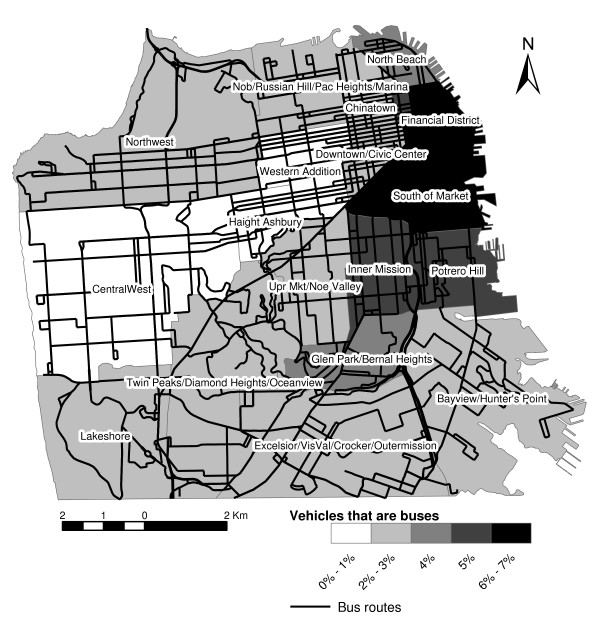
Bus routes and percentage of neighborhood traffic that are buses.

**Figure 9:**
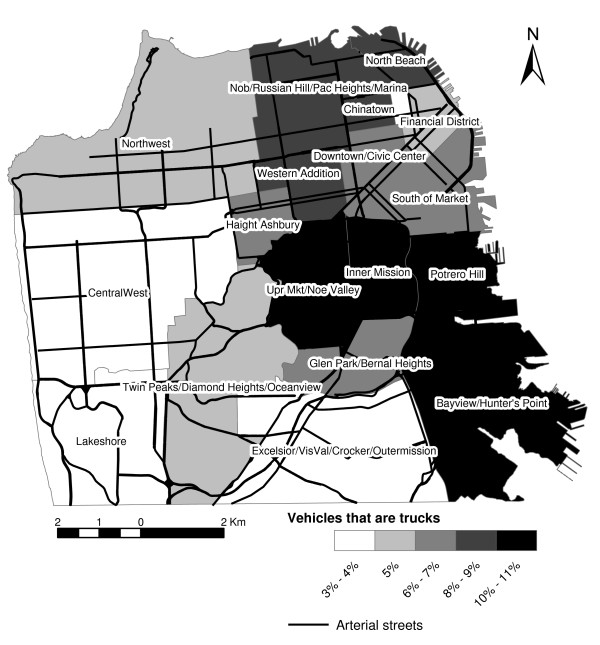
Arterial streets and percentage of neighborhood traffic that are trucks.

Maps of bus and truck percentages (Figures 8 and 9), may help transportation planners understand how these major contributors to noise vary by neighborhood. They indicate how the density of bus routes and the presence of the bus terminal resulted in buses being a major contributor to noise in the Financial District and SoMa neighborhoods. In contrast, the presence of the main postal distribution annex, recycling center drop-off, and numerous light manufacturing made trucks more prevalent in areas like Bayview/Hunter's Point and Potrero Hill, in contrast to streets in the Inner Mission and Upper Market/Noe Valley that were more populated by medium trucks. SoMa also had a high percentage of heavy trucks, again reflecting its major role as a way in and out of the city. Surprisingly, the relatively isolated North Beach area also had high percentages of heavy trucks. However, overall it had much less traffic than SoMa.

Based on the traffic extrapolations to the entire city (Table 2), we find that larger communities had more streets, and hence larger total amounts of community traffic. However, of primary importance is the average traffic per street (Table 1), particularly the street in front of the residence, since the noise-annoyance relationships are based on residential exposures and in open air, traffic noise decreases rather rapidly (6 dB per doubling of distance) [42]. Hence, a large community like the Northwest district had the highest cumulative amount of traffic, but the average noise levels on its arterial and non-arterial streets were relatively low at 68 and 61 dB, respectively.

Although the standard deviations for arterial and non-arterial traffic within each neighborhood were large, arterial streets generally experienced the highest traffic (Table 1), producing noise averaging 68 dB (Table 2). On average, we estimated that 21% of noise exposures along arterials resulted in high annoyance (HA), a relative risk for high annoyance of 1.4 (95 % CI: 1.3–1.5) compared to non-arterials (62 dB, 15% HA). The noisiest community as a whole was SoMa (Table 3, 70 dB, 27% HA). The relative risk of high annoyance in SoMa was 2.1 times higher than the lowest risk neighborhood (Glen Park/Bernal Heights, 61 dB, 13% HA, 95 % CI RR: 2.0–2.2). SoMa was the noisiest neighborhood largely due to high traffic along both arterial and non-arterial streets. In contrast, arterial streets in the Financial District were the noisiest in the city. Although the noise levels may be higher in the Financial District, its taller building heights may reduce exposure and annoyance levels. Moreover, on average the Financial District did not have as high noise as SoMa because the non-arterial streets in the Financial District were relatively quiet.

There were several limitations encountered in our study. One was the difficulty in extrapolating traffic from measured streets to unmeasured ones. Although there were clear differences between arterial and non-arterials, there remained considerable residual variability between streets of the same type and neighborhood. Future improvements to the model might include better categorization of roads, and using more sophisticated traffic flow modeling and/or geostatistical interpolation models. The other limitation concerns the validity of the exposure-annoyance relationship for this present setting. Although the relationship has been shown to be robust across several countries, no US-based studies have contributed to the most recent model [17]. This again highlights the lack of community noise studies in recent US history. Yet an older US-based model exists [13] and shows consistency with the recent models. Still, these models may not adequately account for building age and quality and noise insulating factors that are specific to San Francisco that may modify exposures to environmental noise. In urban environments the degree to which building heights affect exposures may also need to be better considered. It is hoped that this analysis will motivate renewed interest in conducting epidemiological studies on the effects of community noise within the US to develop more specific exposure-outcome models and improved estimates of noise burdens.

It was important to validate the noise predictions from TNM in an urban setting. A regression analysis suggests that the model may have underestimated noise for most noise levels found in community settings. One possible explanation for this may be greater acoustical reflections in an urban landscape, which might not be representative of the freeway settings in which the TNM model was originally developed. Accounting for variations in traffic speed, starting and stopping of traffic, and elevation changes within the city may also explain the underestimate of noise. Correspondingly, the 17% rate of high annoyance for the city may be a conservative estimate. Furthermore, traffic noise is only one component of community noise. A more comprehensive noise assessment in the future might also consider the added noise of living near airports, fire stations, hospitals with emergency vehicles and helicopters, entertainment districts, and various types of rail lines that serve the city. Conversely, various factors can serve to reduce noise which might be considered, including noise attenuation via green spaces, sound barriers, and living in taller buildings that distance certain populations away from street-level noise. Previously, we mentioned how new buses in the city are quieter. We may also find that newer vehicles, such as hybrid/electric vehicles that may be quieter in urban settings may also lower traffic noise.

Despite these limitations, several policy implications emerge from these analyses. The first relates to building design and construction in new urban neighborhoods. Future development of residences in areas of existing high traffic areas should not only be evaluated in terms of the added traffic burden, but on the placement in persons where they will certainly have the potential adverse health impacts. SoMa is a perfect example of such an area, where noise mitigation measures should be employed to protect new residents and thoroughly evaluated. This is also an important consideration for new transit-oriented high-density developments. For instance, new smart growth policies that include a greater reliance on mass transportation, less segregation of land uses, higher residential and commercial density, and complete, mixed-income neighborhoods may have regional benefits that include reducing sprawl and automobile-reliance. However, the benefits of such development need to be balanced against environmental health concerns of having more people live in urban areas of high traffic, noise, and air pollution.

The second implication of this work relates to mitigating traffic demand from new development. In San Francisco over 90% of the city's traffic is due to automobiles, and not trucks or buses. Even though buses and trucks are much noisier than automobiles, with the vehicle type percentages present in San Francisco, automobiles are still the major source of urban noise (e.g., given 1,000 vehicles, automobiles would contribute 64 dB, medium trucks 60 dB, heavy trucks 61 dB, and buses 59 dB, respectively based on hourly LEQs using the TNM assumptions described in Methods). Hence the promotion of walking, bicycling, public transportation, carpooling, work-at-home and telecommuting all equate to less urban noise, and reduced health impacts. If there are good transit options that reduce vehicular traffic, transit-oriented high-density development may reduce noise annoyance. Changes in parking supply and congestion pricing may also be effective measures for reducing traffic demand.

The third implication of this study relates to environmental justice. While some individuals who are highly sensitive to noise may have the means to avoid living in noisy areas, not everyone can afford to live in relatively quieter neighborhoods. Moreover, some highly sensitive individuals may also bear a greater burden of risk, such as elderly persons and children who may be more exposed to daytime noise. Not only should new residents be protected from poorly planned new development, consideration should also be made towards populations currently experiencing the greatest burden of risk. Although the greatest risk currently exists in SoMa, its population is still relatively small compared to those living in other neighborhoods. Our predictions suggest that most of the city's highly annoyed do not live in SoMa. In fact, the highest population densities of highly annoyed exist in Chinatown and Civic Center (average noise levels of 67 and 69 dB, respectively). Hence, if the city were to mitigate noise, for instance by renovation of old construction, it may be most prudent to focus on these neighborhoods, where the most people per area would benefit.

Continued analyses of noise exposure may better elucidate the relationship between the spatial patterns observed and their impacts on low income, different ethnic populations, and children, particularly for these high density areas. In the US, noise issues are typically evaluated at the project level as part of the Environmental Impact Assessment process. However, in other countries noise impacts are increasingly evaluated within a Health Impact Assessment (HIA) process that considers more broadly the overall health of communities. The goal of HIA is to analyze and consider the direct and indirect health effects of public policy ranging from urban planning and transportation to agriculture, energy and natural resources management. The GIS-model presented here for San Francisco can serve as an example of one quantitative tool within the HIA toolbox for the US. Considerable work remains to develop quantitative tools for HIA that can account for the numerous transportation-related health effects. Such tools can serve as a way to track the health of a community over time as it develops. Here, we have established a baseline for noise, which is essential for evaluating current and future changes in annoyance. Our estimate of 17% of the population at risk of being highly annoyed by noise is of considerable concern. Such high rates of annoyance highlight the seriousness of the noise problem for US cities.

## Conclusion

In this paper we present a GIS-based model for evaluating the spatial distribution of traffic-induced noise in an urban environment. Applying the model to the City of San Francisco, we find that the potential risk of annoyance is large, and varies considerably between neighborhoods. This work has implications for building design and construction in new urban neighborhoods, particularly urban infill that may increase density in environments with preexisting noise problems. It also highlights the need for transportation alternatives, as automobiles are the major source of community noise. Finally, the work has implications for environmental justice, as we show that areas of high population density suffer disproportionately from the impacts of urban noise. The relatively simple model presented here may be used to evaluate changes in noise exposures and annoyance as one tool in a larger toolbox for Health Impact Assessments of transportation and land use planning.

## Methods

### Spatial database and traffic analyses

The GIS implemented in ArcGIS [43], includes neighborhood, block and parcel boundaries with land use zoning and building heights attributes, as well as 16,090 street segments for the City of San Francisco. Each street segment corresponds to a city block, and is identified by a unique centerline network number (CNN). Of the total CNNs, 2,634 are classified as arterial street segments, roads defined as major thoroughfares for the neighborhood. The SF Department of Transportation has 6,444 traffic counts for 1,999 CNN segments using pneumatic tube counters from 1992 – 2000. These data were only available due to an effort in 2000 to digitize paper traffic records, which has not occurred more recently. The traffic counts were averaged for segments with multiple measurements. Separate counts were measured for each direction of travel. For two-way streets we summed the measurements taken in opposite directions. We assumed a doubling of traffic if measurements were only taken in one direction of a two-way street. Traffic volumes along uncounted streets were extrapolated from measured neighborhood arterial/non-arterial-specific averages.

Hourly traffic volumes were available for 709 measurements, while 24-hour total counts were available for the remaining counted streets. Based on the hourly traffic data, we computed temporal weighting factors, w(k, t) for each neighborhood k as the 24-hour count divided by the hourly count for hour, t. These weighting factors were then used to convert the streets with only 24-hour total count data to hourly counts. Thus, with hourly traffic estimates for all streets, we were able to compute hourly noise levels as described below.

Traffic noise is largely a function of the vehicular makeup of the traffic. However, data on truck and bus percentages for individual roads were not available for the city. Instead, we derived these percentages using remote sensing techniques. We used an August 2001 georeferenced mosaic of 254 quarter-foot resolution aerial orthophotography, with a positional accuracy of 2.5 feet. From these we performed manual counts of 100 vehicles along arterial and 100 vehicles along non-arterial streets separately for each of the eighteen neighborhoods. Parked vehicles were not counted. Each counted vehicle was classified as an automobile, medium truck, heavy truck, or bus, and used to compute the vehicle makeup by neighborhood and by arterial/non-arterial street status. We further restricted bus fractions to bus routes since this information was available from the GIS. An automated object-oriented classification of vehicle quantity and type from orthophotos is in development [44].

### Noise exposure assessment

The relationship between traffic and noise was assessed via both modeling and field measurement. Various country-specific models are available that model the noise induced by vehicle traffic [45]. In the USA the Federal Highway Administration's (FHWA) Traffic Noise Model (TNM) 2.5 model [41] is generally accepted for estimating traffic-induced noise. Using TNM, we assumed vehicle speeds of 50 km/hr over hard surfaces with receivers located 10 m from the center of the roadway. For simplicity, we did not consider motorcycles, barriers, or reflections other than the hard road surface in the model. Using the hourly traffic estimates and the vehicle makeup percentages, we used the model to estimate hour-specific noise associated with each street segment as follows:

LAeq=10×Log10{[TA1000×10LA/10]+[TMT1000×10LMT/10]+[THT1000×10LHT/10]+[TB1000×10LB/10]}
 MathType@MTEF@5@5@+=feaafiart1ev1aaatCvAUfKttLearuWrP9MDH5MBPbIqV92AaeXatLxBI9gBaebbnrfifHhDYfgasaacH8akY=wiFfYdH8Gipec8Eeeu0xXdbba9frFj0=OqFfea0dXdd9vqai=hGuQ8kuc9pgc9s8qqaq=dirpe0xb9q8qiLsFr0=vr0=vr0dc8meaabaqaciaacaGaaeqabaqabeGadaaakeaafaqaaeGabaaabaGaemitaW0aaSbaaSqaaiabdgeabjabdwgaLjabdghaXbqabaGccqGH9aqpcqaIXaqmcqaIWaamcqGHxdaTcqWGmbatcqWGVbWBcqWGNbWzdaWgaaWcbaGaeGymaeJaeGimaadabeaaaOqaamaacmaabaWaamWaaeaadaWcaaqaaiabdsfaunaaBaaaleaacqWGbbqqaeqaaaGcbaGaeGymaeJaeGimaaJaeGimaaJaeGimaadaaiabgEna0kabigdaXiabicdaWmaaCaaaleqabaGaemitaWKaemyqaeKaei4la8IaeGymaeJaeGimaadaaaGccaGLBbGaayzxaaGaey4kaSYaamWaaeaadaWcaaqaaiabdsfaunaaBaaaleaacqWGnbqtcqWGubavaeqaaaGcbaGaeGymaeJaeGimaaJaeGimaaJaeGimaadaaiabgEna0kabigdaXiabicdaWmaaCaaaleqabaGaemitaWKaemyta0KaemivaqLaei4la8IaeGymaeJaeGimaadaaaGccaGLBbGaayzxaaGaey4kaSYaamWaaeaadaWcaaqaaiabdsfaunaaBaaaleaacqWGibascqWGubavaeqaaaGcbaGaeGymaeJaeGimaaJaeGimaaJaeGimaadaaiabgEna0kabigdaXiabicdaWmaaCaaaleqabaGaemitaWKaemisaGKaemivaqLaei4la8IaeGymaeJaeGimaadaaaGccaGLBbGaayzxaaGaey4kaSYaamWaaeaadaWcaaqaaiabdsfaunaaBaaaleaacqWGcbGqaeqaaaGcbaGaeGymaeJaeGimaaJaeGimaaJaeGimaadaaiabgEna0kabigdaXiabicdaWmaaCaaaleqabaGaemitaWKaemOqaiKaei4la8IaeGymaeJaeGimaadaaaGccaGLBbGaayzxaaaacaGL7bGaayzFaaaaaaaa@8BD9@

where the 1-hour A-weighted sound level (L_Aeq_) is described by LA, LMT, LHT, and LB noise contributions from automobile, medium truck, heavy truck, and bus traffic (T_A_, T_MT_, T_HT_, T_B _in thousands), respectively. Under the assumptions above, LA, LMT, LHT, and LB are 64.0, 73.5, 77.5, and 74.7 dB, respectively.

To validate the model, we choose 235 segments (stratified by neighborhood and traffic level) for field measurement. Freeway on/off-ramps were excluded for safety reasons. Sites were visited between 09:00 – 18:00 hr on weekdays during the summer of 2005. Fifteen-minute L_Aeq _measurements were obtained at each site using Quest Model 1800 Sound Level Meters, and were compared to the results of the TNM model.

In order to create city-wide maps of noise for exposure assessment, we extrapolated the existing traffic count data to the uncounted streets as described above, and then applied the TNM model to compute the hourly noise for each CNN. In accordance with standard community noise assessments, we summarized the hourly measurements into a 24-hour noise indicator, the L_dn_, which applies a 10 dB penalty to noise during the night hours of 22:00–07:00.

### Noise-annoyance assessment

We applied the Miedema and Oudshoorn exposure-response equation for L_dn _and percentage "highly annoyed" (HA)[18]:

%*HA *= 9.994 × 10^-4 ^(*L*_*dn *_- 42)^3 ^- 1.523 × 10^-2 ^(*L*_*dn *_- 42)^2 ^+ 0.538(*L*_*dn *_- 42)

Block-level population data from the 2000 US Census were overlaid upon the modeled traffic noise and used to estimate populations at risk of high annoyance to traffic noise. This was done by buffering each census block 50 m, and taking the mean probability of high annoyance for all streets that are intersected by the buffer. The mean probability was then multiplied by the population living in the census block to estimate the number of highly annoyed.

### Statistical analysis

Linear regression models were used to estimate the relationship between sampled noise levels at a given hour from log-transformed hourly-adjusted traffic counts based on the w(k, t) factor described above, adjusting for neighborhood differences in vehicle makeup. An additional linear model was used to compare TNM predicted noise levels to the field noise measurements. Model error was explored for spatial autocorrelation, and for correlation with time of day. Stata 8.0 was used for statistical analyses [46].

## Competing interests

The author(s) declare that they have no competing interests.

## Authors' contributions

ES and TR designed the study. ES was involved in data collection, GIS and statistical analysis and modeling, and writing of the manuscript. AH worked on the traffic database and analysis of orthophotos. TR and RB contributed to the writing of the manuscript, and were instrumental in interpreting the results in the context of Health Impact Assessment and development trends within City of San Francisco. All authors read and approved the final manuscript.
